# The impact of an online Facebook support group for people with multiple sclerosis on non-active users

**DOI:** 10.4102/ajod.v3i1.132

**Published:** 2014-11-21

**Authors:** Jacqui Steadman, Chrisma Pretorius

**Affiliations:** 1 Department of Psychology, Stellenbosch University, South Africa

## Abstract

**Background:**

Multiple sclerosis (MS) is a debilitating disease and there is little research on support networks for people with MS (PwMS). More specifically, most studies on online support groups focus on those who actively participate in the group, whereas the majority of those who utilise online support groups do so in a passive way.

**Objectives:**

This study therefore aimed to explore the experiences of non-active users of an online Facebook support group for PwMS. Emphasis was placed on the facilitators and the barriers that were associated with membership to this group.

**Method:**

An exploratory qualitative research design was implemented, whereby thematic analysis was utilised to examine the ten semi-structured interviews that were conducted.

**Results:**

Several facilitators were acquired through the online support group; namely emotional support (constant source of support, exposure to negative aspects of the disease), informational support (group as a source of knowledge, quality of information) and social companionship (place of belonging). Some barriers were also identified; namely emotional support (emotions lost online, response to messages, exposure to negative aspects of the disease), informational support (information posted on the group, misuse of group) and social companionship (non-active status)

**Conclusion:**

These findings demonstrate that the non-active members of the online support group for PwMS have valid reasons for their non-active membership status. More important, the findings suggest that the online Facebook support group provided the group members with an important support network in the form of emotional support, informational support and social companionship, despite their non-active membership status or the barriers that have been identified.

## Introduction

Multiple sclerosis (MS) is a chronic inflammatory and degenerative disease that affects the central nervous system (CNS) (Boeschoten *et al*. 2012). MS is a relatively common disease, with a global prevalence rate that is on the rise (Young 2011). In a 2008 study conducted by the World Health Organization and the Multiple Sclerosis International Federation, it was estimated that the global prevalence rate of MS was 30 per 100 000 (Dua & Rompani 2008). The symptoms of MS are different for every person, depending on the location of lesions and the extent of damage in the CNS, and symptoms often change over time as the disease progresses (Mozo-Dutton, Simpson & Boot 2012). The most common symptom of MS is fatigue, which often results in loss of employment and can lead to self-imposed social isolation (Grytten & Måseide 2006). The impairments associated with MS often result in limited physical activity and restricted social participation (Uccelli *et al*. 2004). At present there is no cure for MS; however, there are medications that are able to slow down the progression of MS and ease its symptoms (Reipert 2004). Psychological treatment is important for people with MS (PwMS) due to the high levels of anxiety and depression among these individuals (Chalk 2007). Though psychological treatment assists individuals to physically and mentally cope with MS, it has been found that social support assists individuals to cope emotionally (Pretorius & Joubert 2014; Schwartz & Frohner 2005). Social support is thus an important resource for those with MS, as it often increases self-esteem, decreases depression and increases quality of life (Pretorius & Joubert 2014; Schwartz & Frohner 2005). Peer support group programmes have become a popular means of providing social support to individuals who share similar difficulties, despair, diseases, and pain (Uccelli *et al*. 2004). However, the apparent lack of research on suitable support networks for PwMS, such as peer support groups, is concerning, since the disease has such a debilitating impact on the lives of so many individuals.

Traditionally these peer support groups consisted of personal face-to-face meetings where people have the opportunity to share their experiences and knowledge and to give and receive emotional support (Droge, Arnston & Norton 1986). These face-to-face support groups typically comprise of people who have a diagnosis in common and operate on the premise that sharing information with individuals in similar situations can be beneficial without the presence of a healthcare professional (Droge *et al*. 1986). While one of the goals of face-to-face MS support groups is to improve psychological health, this does not seem to be the case (Wakefield, Bickley & Sani 2013). For example, Uccelli *et al*. (2004) found that face-to-face support groups did not decrease levels of depression or increase quality of life for PwMS. Wakefield *et al*. (2013) conducted a study from a social identity approach and suggest that it is the level of subjective identification with a support group (rather than simply support group membership alone) that affects the mental health of PwMS in a positive way. Healthcare professionals often encourage MS patients to make use of face-to-face support groups, and there are MS societies across the globe who provide financial and human resources to organise, implement and maintain these groups (Finlayson & Cho 2011). However, limited studies have sought to investigate these MS support groups in terms of who utilises them, who no longer utilises them, and who desires to participate in them (Finlayson & Cho 2011).

The use of online support groups is becoming more popular as the technology develops. An online support group is a type of support group that an individual can access on the Internet through the use of any type of computer-mediated communication, such as a mobile phone, computer or laptop. It has been reported in 2007 that 36 million people in the United States of America (USA) are members of some or other online support group (Coulson, Buchanan & Aubeeluck 2007). Facebook is an example of one of the most popular places where online support groups can be found. Online support groups enable individuals to engage in supportive interaction using listings, chat rooms, bulletin boards and personal email exchanges with others who share similar problems or challenges (Barak, Boniel-Nissim & Suler 2008; Coulson *et al*. 2007). Members of online support groups often utilise these groups to exchange different kinds of support, and studies suggest that informational as well as emotional support are the most frequently provided (Coulson 2005; Ravert, Hancock & Ingersoll 2004). Network support also emerges, whereby members use the support group as a common meeting ground and where all issues relating to a specific illness or problem can be discussed (Coulson *et al*. 2007). Although the Internet is nowadays an important source of information and emotional support for many people, there seems to be a paucity of research focussing on online support groups. Research that has addressed the impact of online support groups thus far have focussed on more well-known conditions such as cancer, arthritis, Parkinson’s disease, Huntington’s disease, irritable bowel syndrome, fibromyalgia and human immunodeficiency virus/acquired immunodeficiency syndrome (HIV/AIDS) (Attard & Coulson 2012; Coulson 2005; Coulson *et al*. 2007; Høybye, Johansen & Tjørnhøj-Thomsen 2005; Mo & Coulson 2010; Van Uden-Kraan *et al*. 2008b), whilst more uncommon diseases such as MS have received little attention (Coulson *et al*. 2007).

Several studies have found that those who utilise online support groups benefit in several ways; however, most studies on online support groups have focused on those who actively participate in the group, whereas the majority of those who utilise online support groups do so in a passive way (ranging from 45.5% – 90% of group members) (Buchanan & Coulson 2007; Coulson 2005; Nonnecke, Andrews & Preece 2006; Sun, Rau & Ma 2014; Van Uden-Kraan *et al*. 2008a). It is clear from the literature that descriptions of non-active members of online support groups (also known as lurkers) vary. Dictionary.com (n.d.) defines a lurker broadly as: ‘One of the “silent majorities” in an electronic forum, one who posts occasionally or not at all, but is known to read the group’s postings regularly.’ Some studies define non-active members (lurkers) as the members who never post in an online group (Neelen & Fetter 2010; Nonnecke *et al*. 2006), or members who post messages only once in a while (Golder & Donath 2004). Other studies refer to non-active members (lurkers) as the members who post three or fewer messages from the beginning or users who never posted messages in the last four months (Ganley, Moser & Groenewegen 2012). It seems as if the main difference between active members (posters) and non-active members (lurkers) is that active members (posters) make contributions to the group by posting regularly, while non-active members (lurkers) stay silent most of the time. A recent literature review by Sun *et al*. (2014) emphasises that different methods exist to identify and describe non-active members of online groups (lurkers) and depend on the nature of the online group and the purpose of the study.

At present it is unclear whether non-active members benefit to the same extent that active members do, as there have been limited studies on this topic and results have been inconclusive (Nonnecke *et al*. 2006; Sun *et al*. 2014; Van Uden-Kraan *et al*. 2008a). A study that examined the differences between empowerment outcomes for active and non-active posters of online support groups for individuals with fibromyalgia, breast cancer and arthritis was conducted by Van Uden-Kraan *et al*. (2008b). The findings indicated that non-active members tend to benefit in similar areas compared to the active members of support groups. Mo and Coulson (2010) found that non-active members in an online support group for HIV and AIDS did not differ from active posters in their levels of care, self-efficacy, optimism, depression, and loneliness. They also found that non-active members felt more energetic than active posters. In contrast, Barak *et al*. (2008) found that active members experience greater emotional relief compared to non-active members.

The available literature suggests that there are several advantages of online support groups (Coulson *et al*. 2007). These groups are not subject to spatial, geographical and temporal constraints; participants are able to post messages at their own pace. These groups bring together a variety of individuals, offering different perspectives, experiences and opinions. Furthermore, participation on an online support group affords the individual more anonymity than face-to-face support groups (Coulson 2005; Finn 1999; White & Dorman 2001). Online support groups also allow a learning opportunity for new members, relatives, professionals and friends (Finn 1999). Even the non-active members of the support group (those who do not participate often, but read messages regularly) are able to benefit from the group without disrupting the group process (Finn 1999). Members of online support groups frequently use these groups to exchange support and research suggests that emotional and informational support are the most common forms of support reported by group members (Coulson 2005; Malcomson, Lowe-Strong & Dunwoody 2008; Ravert *et al*. 2004; Van Uden-Kraan *et al*. 2008b). Online support groups do not only seem to contribute to the empowerment of individuals with chronic illnesses, but also result in a decrease in social isolation and facilitate new social networks (Høybye *et al*. 2005; Malcomson *et al*. 2008).

On the other hand, the disadvantages of using online support groups are that they may take time away from face-to-face social contact and users may become dependent upon them (Barak *et al*. 2008; Finn 1999). The use of online resources is often only available to those with computer skills and access to computers, which excludes the poor, undereducated and possibly the elderly (Finn 1999). Furthermore, a study conducted by Im *et al*. (2007) found that members of online support groups are often a select group consisting of white, middle-aged, middle class, well-educated individuals. There is also concern regarding the quality of information being provided by members of the online support group (Finn 1999; Van Uden-Kraan *et al*. 2008a).

It is clear from the literature that limited research has been conducted on face-to-face support groups for people with MS (Uccelli *et al*. 2004), while there appears to be a paucity of research focussing on online support groups for PwMS and even less research on the non-active users in these groups. As discussed, Uccelli *et al*. (2004) found that traditional face-to-face support groups did not make a significant contribution to a decrease in depression or an increase in the quality of life in PwMS. A possible explanation for this is that, although PwMS identify social support as an important resource, this type of support may not always fulfil the needs of PwMS. PwMS face many challenges that can make access to face-to-face support groups difficult. These challenges relate to the symptoms that these individuals present with and can include problems with mobility and fatigue, cognitive impairments and problems with bladder and bowel control. According to Coulson *et al*. (2007), it is possible that online support groups can provide individuals with chronic illnesses with the same type of support as face-to-face support groups, but without the potential challenges that have been identified. When the existing literature regarding the advantages of online support groups is considered, it can be speculated that the non-active members of the online support group for PwMS, which comprise the majority of the support group, may benefit from the online support group in different ways. The online support group can potentially address the physical problems with fatigue and mobility that are commonly reported by PwMS, because these individuals can be part of an online support group in the comfort of their own home without any geographical boundaries. It is also possible that membership of this group could address the need for the emotional support and social isolation that is commonly reported by these individuals. Due to the paucity of research on online support groups for PwMS, the above-mentioned examples of potential benefits for these individuals are based on speculation when the number of challenges that PwMS face and the benefits of online support groups are considered (Coulson *et al*. 2007; Finn 1999; Høybye *et al*. 2005; Ravert *et al*. 2004; White & Dorman 2001). Furthermore, given the exponential growth in the use of online support groups, it is important to gather accurate information about the quality and value of this type of support for PwMS. This study will hopefully contribute to the limited knowledge that is currently available on this topic.

Therefore, the aim of this exploratory study was to examine the experiences of non-active members of an online Facebook support group for PwMS by exploring the facilitators and barriers that are associated with membership of this group. A facilitator refers to any factor that makes a process or situation easier to deal with, while a barrier has been defined as any situation or obstacle that needs to be overcome in order to make progress (Stevenson 2010). For the purpose of the present study, a facilitator will encompass any support, services, or processes that are helpful to the participants of this study, while a barrier will refer to any aspect that makes the experience of being part of the online Facebook support group challenging.

## Method

### Research design

An explorative qualitative research design was utilised to investigate the impact of an online support group for MS on non-active users. Individual, in-depth semi- structured interviews were implemented as the data collection method.

### Participants and procedure

The participants were 10 individuals who were all part of an online Facebook support group for PwMS. The sample consisted of 10 females, aged between 28 years and 55 years (Median = 50). The duration since MS diagnosis ranged from 2 years – 25 years (M = 12.65; SD = 8.77), and the period of membership of the online support group ranged from 2 months to 5 years (M = 2.12; SD = 1.07). There were vast differences between participants in the years since diagnosis as well as the duration of membership of the online support group, which resulted in a heterogeneous sample. MS is a complex disease that is diverse in nature, and it impacts various individuals in different ways. It thus seemed fitting to examine the experiences of a heterogeneous sample that might be a more adequate representation of the broader population of PwMS who utilise online support groups.

Participants were identified with the assistance of the chairperson for the MS Society of the Western Cape (South Africa), who took the initiative to start the online Facebook support group and currently manages the administration of the group. A message was posted to the group explaining the objectives of the study. Members of the online support group who were interested in participating in this study were invited to indicate their interest to participate either to the chairperson of the MS Society, or to the researchers via email. The inclusion criteria for participants of this study were that: (1) they had to be non-active members of the support group (members who post messages occasionally or not at all) and (2) they read the group’s postings regularly. Ethical approval has been granted from the Health Research Ethics Committee at the university (Ethics Reference Number S13/04/074).

Individuals who indicated that they were willing to participate in the study were contacted via telephone or email to arrange a meeting with each participant at a time and place of their choice. All the interviews were conducted in the participant’s home. Data collection began with explaining the aims of the study, confidentiality of data, and the rights of the participants, and informed consent was sought from the participants. The participants were then requested to provide basic biographical information, such as their gender, age, duration of illness, as well as how long the participant has been part of an online support group. Thereafter a 60–90 minute individual, in-depth semi-structured interview was conducted with each participant. These interviews were guided by the following questions:

- What types of support networks are available to you?
- What types of support do you get from the online support group?
- What facilitators are provided by the online support group?
- Are there any barriers involved in being part of an online support group?

To assist the transcription process, each interview was audio-recorded with permission from each participant.

### Data analysis

Data were analysed by means of a qualitative method known as thematic analysis. Thematic analysis is a method for discovering, examining and recording patterns within a data set (Braun & Clarke 2006). According to this method, the first step entails researchers familiarising themselves with the data, which is achieved by transcribing the data followed by reading and re-reading it until they are able to generate initial ideas from it (Braun & Clarke 2006). The second step involves generating initial codes from the data, which involves identifying aspects of the data that appear interesting; thereafter, data is collated according to each code (Braun & Clarke 2006). Step three involves sorting through the codes to identify potential themes and then combining these coded extracts to form an overarching theme (Braun & Clarke 2006). The fourth step is to review identified themes, which involves establishing whether potential themes correlate to the coded extracts and then to the entire data set (Braun & Clarke 2006). Step five involves naming and defining each theme to refine it; this entails writing a detailed analysis for each theme and then identifying where the theme fits into the overall description of the data (Braun & Clarke 2006). The final step is to write a report. This write-up must provide vivid examples that validate the argument that is being made, and this can be achieved by using direct quotations from the interviews to demonstrate the identified themes (Braun & Clarke 2006).

### Contextualising the findings

Social support has developed into an umbrella term that refers to the various aspects involved in social relationships, and in most cases it refers to functions that are performed for an individual by their significant others (Schwarzer & Leppin 1988). Researchers have found that an ideal measure of social support was to examine subjective experiences of perceived functional support (Cohen & Wills 1985; House & Kahn 1985). Functional support can thus be broken into its components in order to evaluate how each component relates to different outcomes (Sherbourne & Stewart 1991). The framework that was used to make sense of the results of this study was the five components of functional support, namely emotional support, instrumental support, informational support, appraisal support and social companionship (Sherbourne & Stewart 1991). This framework was chosen because an online support group provides a form of support and this study aimed to explore the types of support that are acquired and the barriers that are associated with non-active members of this group.

Emotional support refers to the acquisition of love, care, esteem and empathy from others (Schwarzer & Leppin 1988). Instrumental support involves the provision of physical or mental assistance from others when facing a task. This form of support can be material support, financial assistance or services and encompasses the direct ways that individuals help others (Schwarzer & Leppin 1988; Sherbourne & Stewart 1991). Informational support involves the provision of knowledge to enable understanding and coping with a particular situation (Cohen & Wills 1985; Schwarzer & Leppin 1988). Appraisal support involves the validation of an individual’s cognitions, beliefs or emotions regarding a situation or their self (Schwarzer & Leppin 1988). Social companionship involves sharing leisure time, laughing, dining out, conversing or collaborating together (Schwarzer & Leppin 1988).

The facilitators and barriers that were identified in this study comprised mainly of emotional support, informational support and social companionship.

### Ensuring rigour

There are various means to establish rigour in qualitative research and several of these methods were utilised to ensure the rigour of this study: namely reflexivity, member checks, and peer debriefing. Reflexivity necessitates that the researcher reflects on their own beliefs in the same manner as they examine the beliefs of their participants (Krefting 1991). The primary researcher enhanced reflexivity by discussing emergent findings with the project leader, who has knowledge of MS and experience of qualitative research. Member validation (or checks) involves checking the findings of the collected data with the members of the participant group (Long & Johnson 2000). This process was undertaken during data collection, where the primary researcher confirmed points that were made by participants during the interviews. Peer debriefing can be pursued by discussing emergent findings at regular intervals with knowledgeable colleagues. This stimulates exploration and consideration of additional explanations and perspectives at different stages of data collection and analysis (Long & Johnson 2000). This method was utilised by discussing and comparing ideas, methods, and findings with the project leader throughout the research process.

## Results

It was evident from the interviews with the participants that, despite differences in the duration of membership of the online support group as well as variation in the time elapsed since MS diagnosis, there were several themes that appeared to be common across the experiences of participants. As discussed earlier, the results of this study were interpreted according to the model of functional support which comprises of five types of support (emotional support, informational support, social companionship, instrumental support and appraisal support) (Sherbourne & Stewart 1991). The key themes and their respective sub-themes that were identified through the process of thematic analysis can be found in Table 1. Each theme possessed various sub-themes that could be classified as facilitators (any support, services, or processes that are helpful to the participants) and/or barriers (any aspects that make the experience of being part of the online Facebook support group challenging) that were associated with membership of an online support group. It is vital to note that these themes are not displayed or discussed in any particular order of significance.

**TABLE 1:** Themes and their respective sub-themes that were identified during thematic analysis.

Thematic Analysis	Main theme	Sub-theme
Facilitators	Emotional support	Constant source of support
		Exposure to negative aspects of disease
	Informational support	Group as a source of knowledge
		Quality of information
	Social companionship	Place of belonging
Barriers	Emotional support	Emotions lost online
		Response to messages
		Exposure to negative aspects of the disease
	Informational support	Information posted on the group
		Misuse of group
	Social companionship	Non-active status

### Facilitators

With regard to the facilitators that were accessible through membership of an online support group, three main themes were identified, namely emotional support, informational support and social companionship.

#### Emotional support

The first main theme that was identified during data analysis was that participants acquired significant emotional support through belonging to the online support group despite their non-active membership status. The sub-themes identified as emotional facilitators were: constant source of support and exposure to negative aspects of the disease.

##### Constant source of support

The majority of the participants reported that although they regularly read the messages that are posted in the group, they hardly ever post messages. Regardless of this, several participants indicated that the group was a constant source of emotional support for its members. One participant said the following: ‘… with this group I know I will never be alone again because they are there for me and when I know an answer I’m there for them’ (P6).^1^

The online support group was also a source of understanding and genuineness/empathy for certain participants, as illustrated by the following extract:

‘You know what makes me feel good is that they have such genuine comments, they don’t know this person from a bar of soap but they’ve taken the time to write something.’ (P2)

Participants also noted that emotions were sometimes conveyed through messages despite communicating through an online medium, as individuals often felt connected to others without directly communicating with them:

‘I will always go on there to read the messages, it is like my family; it’s like real close friends even though I’m not in a personal way close to them.’ (P7)

##### Exposure to negative aspects of the disease

Participants indicated that exposure to the negative aspects of the disease often served as a reality check for them, as it caused them to feel grateful for their health:

‘I’m also grateful for it because I’m very fortunate not to be as bad as lots of people. I see what everybody is going through…it really makes me feel so blessed.’ (P10)

The participants also seem to admire the coping skills of group members who are much worse off that what they are:

‘I think it’s good that there are people in the group who are progressed quite far. Like this one lady is in a wheelchair, but she’s magnificent. You know what, she never ever complains and to me those people are the people that I think ‘Wow’.’ (F3)

#### Informational support

Another main facilitator that was identified through the online support group was informational support. Two sub-themes were identified in relation to this form of support, group as a source of information, and quality of information.

##### Group as a source of information

The online support group often served as a source of information for many participants:

‘You see the agony of people trying to go through getting this needle to go into their skin and they share this on the system and then you get the tips about, ‘Well, I rub apple cider vinegar onto the soft side because it helps with the irritation of the site’, the other one says, ‘My husband’s learnt to do the injections’. That is wonderfully encouraging.’ (P2)

Participants also noted that the information provided in the online support group often improved their knowledge regarding MS: ‘The online support group, it offers a lot of research and it actually educates you more about the illness’ (P6).

In addition:

‘It’s nice to hear what other people are going through, and it teaches you more about MS as well because there are things that you don’t know and then you can hear what they are doing.’ (P10)

##### Quality of information

Participants noted that the information provided by the online support group was of a good quality, especially when it was provided by the older members or professionals of the group who had experience with MS and could thus offer practical advice to other members:

‘There are a lot of people that have been having MS for ten, twenty years, and I’ve just had it for six years now, so my knowledge of this is not that good so I prefer the older members to actually give that kind of answers.’ (P6)

The online support group also served as a source of research about MS, where members would post links to articles related to cures, symptoms, and explanations of the illness, which was noted as an empowering resource by certain participants, as illustrated by the following extract:

‘I do read everything in all the links, there is always some extra information out there, and it’s nice to know and be on top of everything. And then it actually makes the whole MS journey light to bear, because if you are informed it takes the fear away.’ (P10)

#### Social companionship

The online support group provided the opportunity for social companionship for several participants, which could be a vital resource for individuals with a disease that often results in isolation.

Place of belonging: Participants indicated that the online support group often served as a place of belonging for them, as it provided a common ground where different individuals could come together to discuss various topics:

‘It is nice to hear about what they say, ‘I’ve just gone for my first injection today and this is how I feel’, and whatever the case may be. Because when I started my medication it was nice to know what the symptoms would be, that type of thing.’ (P10)

### Barriers

Some barriers that accompanied membership of this online support group were also identified during data analysis. Three main themes were identified; namely emotional support, informational support, and social companionship.

#### Emotional support

The first main theme that was identified was that participants experienced several challenges in relation to emotional support. The sub-themes related to the emotional challenges encountered by these individuals were: emotions lost online, response to messages, and exposure to negative aspects of the illness.

##### Emotions lost online

It appears that participants felt that emotions were not always properly conveyed when they were communicated through an online medium. Individuals noted that MS can be an isolating disease, which illustrates the importance for adequate emotional support. Furthermore, participants mentioned that the inability to perceive or express emotions created difficulties when it came to forming connections with other members, as it was not possible to view body language; for example: ‘But, otherwise online sometimes you can’t just express it. With text and emails everything, we kind of develop something where all emotions are cut off’ (P6), and ‘I can’t see their body language. I can’t see their eyes, their eyes to me is the mirrors of your soul. You can’t see the gentleness of the person’ (P7).

A perceived lack of emotional connection meant that individuals did not always feel comfortable enough to share their experiences with others on the online support group, which led to feelings of isolation as participants longed for a human touch that was lacking on the group: ‘Via email and SMS’s you can’t feel that emotional connection with somebody, so it kind of pushes you away emotionally. You feel kind of on your own island’ (P6).

##### Response to messages

It became evident that the ability to communicate on the online support group was often hindered in certain participants due to severe MS symptoms that affected their mobility as well as their capacity to utilise the computer. This could be one explanation of why all the members on the group do not respond to or comment on posts made by others on a regular basis:

‘For me it is the fact that I cannot do postings and participate. I guess I could ask my husband to type in for me, but he does so much for me already, I just don’t want to burden him with frivolous things.’ (P8)

##### Exposure to negative aspects of disease

Another emotionally challenging aspect of online support group membership was that participants were often exposed to negative aspects of the disease through posts made by other members. Common feelings that emerged as a result of exposure to negative aspects of MS being shared on the group were sadness, uncertainty, and feelings of negativity. One participant noted that exposure to negative aspects of MS, such as hospitalisation, relapses, and the death of other members, provoked feelings of sadness, especially during periods of good health:

‘When I look at all the messages on Facebook and I see how the people are suffering and how difficult it is and me also being an MS sufferer, I am doing so well. It actually makes me sad. This one is going into hospital and this one is going for this injection. It makes me sad.’ (P9)

The exposure to negative MS symptoms often aroused feelings of uncertainty for participants, as they were exposed to an array of symptoms that they might not have experienced yet. One participant explained:

It is the negativity, it is when you do see a symptom that maybe you haven’t experienced personally it is the thought that, ‘Oh, is that one still coming my way?’, so there is that exposure. (P2)

Participants also noted that feelings of negativity were aroused in response to complaints or sharing of negative aspects of the disease on the online support group, which was not something that they wanted on the group:

It makes me feel sorry for the people and I realise that I might be there one day and at this point I don’t want to think like that. I want to go for it as long as it is going good, I want to let it last for as long as possible. (P9)

#### Informational support

Another prominent challenge that was identified was deficits in informational support. The online support group served as a vital source of informational support for several participants; however, the following sub-themes indicate how this form of support was impacted on by the information posted on the group as well as the misuse of the online support group.

##### Information posted on the group

Participants also indicated that the amount of links that were posted on the online support group, which would redirect members to articles or sites with information about MS, could be overwhelming at times, especially when individuals were not familiar with the Internet. This challenge was perceived as an overload of information, as described by the following extract:

The internet, some of us didn’t grow up with it. It is overwhelming in terms of the amount of information. There are a lot of good sites and links that they do put there. There is just so much word overload. (P2)

In addition:

I feel that sometimes there’s a bit too much information on there. (P5)

The quality of the information supplied by members of the online support group was also found to be a challenging aspect for certain participants, as they were often uncertain about the accuracy of this information: ‘Sometimes I’m like okay, I’m not sure, but I think I’m going to Google that just to make sure about that because it doesn’t sound kosher’ (P6).

##### Misuse of group

Participants also indicated that the misuse of the online support group was a challenge, as group members would sometimes make posts that were not MS-related, which could challenge the purpose of the group or cause other members to not see important MS-related posts: ‘Sometimes it goes a bit off topic and then you miss the important things’ (P5).

It was also noted that the group had become similar to a chat room, where conversation was not MS-related and the purpose of the group was undermined, as one participant explained:

‘All of a sudden there’s a whole conversation like it’s a chat room. To me it’s not a chat room, it, that doesn’t serve the purpose of what it’s there for and I don’t like that. I would change that people can just do random chatting there because if I want to do random chatting, I phone my best friend.’ (P3)

#### Social companionship

Many of the participants experienced challenges in relation to social companionship. Factors associated with their non-active status were identified as the main influence that hindered participants’ abilities to socialise.

Non-active status: Several participants mentioned that their non-active status on the online support group often influenced their ability to form bonds with other members, as other members did not always keep in contact with them, which made socialisation difficult: ‘Because of that, me not being so active, people don’t ask me just, ‘How are you, what happened, how far is the divorce, how’s your life’, that doesn’t happen’ (P7); and ‘I’m not very active, so I don’t get a chance to build a relationship or a friendship with somebody’ (P9).

## Discussion

Coulson (2005) suggested that it is important to focus on the impact that messages posted online have on recipients, whether it be the intended recipient or those who are non-active in the group. This study focused on the non-active users of the online support group for PwMS, as previous findings suggest that even the non-intended recipients of posts paid attention to comments made by other members, and this information provided by their peers was regarded as a reliable source by those who read it (Coulson 2005; Preece, Nonnecke & Andrews 2004). In line with previous research, it was evident from the findings of this study that there could be many reasons why PwMS are involved in the online Facebook support group in a non-active way. Similar to the findings of Preece *et al*. (2004), several of the participants seem to prefer to be non-active members of the online support group because they acquire sufficient support by merely reading the posts and replies made by other members. On the other hand, MS is known to be a debilitating disease that can create difficulties in physical and motor coordination (Mozo-Dutton *et al*. 2012; Uccelli *et al*. 2004), which would make it difficult to utilise a computer and to post on the online support group. Some participants in this study faced severe mobility difficulties due to MS symptoms that prevented them from participating in the group, despite a desire to be more involved.

Several studies have found that perceived social support is an important resource for individuals with MS, as it often improves their coping strategies in the face of the many challenges that are associated with MS (Chalk 2007; Malcomson *et al*. 2008; Mohr *et al*. 1999). Such findings also emerged in this study, as the participants considered the emotional support, informational support and the social companionship provided by the online Facebook support group for PwMS to facilitate their day-to-day coping with MS, despite their non-active membership status. One of the most vital facilitators was the acquisition of emotional support from members of the online support group, which consisted of sub-themes relating to a constant source of support and exposure to the negative aspects of the disease. The group served as a vital source of emotional support for several participants, as members would provide genuine responses to messages posted on the group by expressing empathy, acknowledging how individuals were feeling, and reciprocating emotions (Coulson 2005). Despite the probability of having very little direct contact with group members due to their non-active status, they were often able to relate to the experiences and emotions expressed in the group. This is possibly because individuals had developed a mutual understanding as other members had already experienced similar emotions and thus had a level of understanding that family members or friends did not (Attard & Coulson 2012). Furthermore, although the participants mainly observed others sharing their experiences (whether positive or negative), without actively sharing themselves, they reported that it gave them the opportunity to see that there were others on the group who were facing more challenging circumstances than they were. This allowed the participants to gain perspective about their illness and this possibly assisted them in attaining a sense of acceptance for their disease (Attard & Coulson 2012; Malcomson *et al*. 2008).

Responses to messages have been identified as a potential barrier to emotional support. It has been reported that individuals with Parkinson’s disease were often unable to type and answer posts as they were hindered by the symptoms of their disease (Attard & Coulson 2012). This appeared to be the case for several participants in the present study, as several individuals noted that their severe physical symptoms, which affected their mobility as well as their capacity to utilise the computer, often prevented them from typing or responding to posts. This could be another explanation of why all the members on the group are not actively participating in the online support group on a regular basis. A previously identified challenge of online support group membership – namely the difficulty of being exposed to negative aspects of an illness – was also found to be a prominent challenge among participants, as it was found to arouse feelings of uncertainty and sadness (Van Uden-Kraan *et al*. 2008a).

Another prominent theme that was identified was informational support, where group members provided a wealth of information relating to topics such as disease management or symptom interpretation, which assisted participants to cope with the various barriers that were associated with MS (Attard & Coulson 2012; Coulson 2005; Coulson *et al*. 2007). It has been speculated that individuals who have been living with MS for longer periods have gained a level of experience that allows them to provide useful information to others (Malcomson *et al*. 2008). Similar findings emerged in the present study, as participants expressed a preference for the advice and insight that was provided by older members of the group. The group also served as a source for research, where individuals would have access to the latest information on cures and medications for MS. Previous findings suggest that individuals who were equipped with the latest knowledge of their disease experienced a sense of empowerment (Malcomson *et al*. 2008). Such findings also emerged in the present study, as several participants mentioned that being informed about MS led to a decrease in fear associated with the disease. It was noteworthy that although the participants were non-active and mainly observed and read the messages that were posted, they benefited from the informational support that is provided by the online support group for PwMS.

The most prominent challenge that was associated with informational support encompassed uncertainty regarding the quality of information that was being posted on the group. Findings by Van Uden-Kraan *et al*. (2008b) and Finn (1999) have suggested that individuals often worry about the quality of information being provided on online support groups; however, certain participants noted that the quality of the information posted on the group was high, as it was being supplied by professionals and individuals who had a wealth of knowledge regarding MS. Such individuals have been known to intervene if there was any misinformation being provided on the group (Van Uden-Kraan *et al*. 2008a). Too much posting was also noted as a challenge to informational support, as several participants felt that there was often an information overload on the group, where members would post too many links to the group or too many of the same questions. This mirrors findings made by Van Uden-Kraan *et al*. (2008b) that members would often outgrow their support groups due to the repetition of questions or posts.

One of the most prominent themes among participants was the acquisition of social companionship through the online support group, which consisted of the sub-theme encompassing a place of belonging. The risk of social isolation can be elevated among individuals with MS due to the interference of symptoms with daily functioning or a lack of helpers available (Finlayson & Cho 2011); therefore, it is possible that the online support group served as an important means of preventing social isolation in PwMS. The online support group fostered a sense of belonging among many participants, as it allowed different individuals to meet together and discuss various topics relating to their illness. This facilitator created a sense of empowerment and comfort because individuals no longer felt that they were facing MS alone, as they were possibly aware that they belonged to a network of support that would always be available (Coulson *et al*. 2007). It is remarkable that the participants of this study experienced a sense of belonging to the online support group for PwMS, despite their non-active membership status.

With regard to barriers to social companionship, participants found it challenging to form friendships with other members of the online support group and two participants indicated that their non-active status was the reason behind this. This finding contradicts results of a study by Attard and Coulson (2012) examining communication on an online support group for people with Parkinson’s. Attard and Coulson (2012) found that friendships are usually formed easily in contexts where individuals share similar experiences and feel belonging to a specific group. However, the aforementioned study examined a larger sample of active posters, which might account for this discrepancy.

### Limitations and directions for future research

Firstly, the findings of this study could be limited by the fact that a small sample size was used consisting primarily of individuals who resided in the Western Cape, which limits the ability to generalise the results of this study to the broader population of individuals with MS in South Africa who utilise the online support group. It would be beneficial to replicate this study among a more representative sample that would encompass individuals from different regions in South Africa.

Secondly, the participants of this study consisted of individuals who are non-active users of the online support group; it is thus possible that the participants are not representative of all participants who belonged to the MS online support group. This broad representation was never the aim of this study. It is however recommended that future studies explore comparisons between active and non-active users to examine whether the two groups differ in their experiences of the online support group.

Thirdly, it should be noted that the findings are based on the subjective experiences of a once-off semi-structured interview with the participants. Participants themselves estimated to what extent they benefited from the online support groups. Although this study provided us with relevant insights into the impact of an online Facebook support group for PwMS on non-active members, a longitudinal study would be useful in evaluating whether the non-active group members truly benefit from the online support group.

Finally, it was evident from the findings of this study that the participants experienced a sense of belonging to the online support group for PwMS. Future research could investigate the extent to which PwMS identify with their online support group, such as feeling a sense of belonging to the group and a sense of commonality with other members of the group, and the implications this has for their well-being. This could contribute to the research conducted by Wakefield *et al*. (2013) who investigated this issue in the context of a face-to-face MS support group.

## Conclusion

Two key conclusions can be drawn from this study. First, it is evident that the non-active members of the online support group for PwMS have valid reasons for their non-active membership status. This seems to support the suggestions of previous research that non-active membership status in an online support group should be viewed not only as normal, but as a valuable and valid form of online behaviour. Second, and most important, the findings suggest that the online Facebook support group provided the group members with an important support network in the form of emotional support, informational support and social companionship, despite their non-active membership status or the barriers that have been identified.

Regarding the practical implications, the physical challenges that have been identified as a major barrier for some could be addressed by the creation of more accessible and user-friendly patient-oriented websites and platforms. With regard to the lack of social companionship, the challenge for health professionals is to understand how the non-active experience can be more effectively supported to increase feelings of membership with the online Facebook support group for PwMS. This could also be addressed with the assistance of organisations such as the MS Society of the Western Cape by finding creative ways to involve not only the participants who are generally actively involved on the online support group, but also the non-active members. Last, but most important, health professionals should encourage PwMS to join online support groups, since active as well as non-active members of these groups seem to benefit from support that is provided by online support groups.

## Footnotes

1 Note: Participant code: P = participant; 1 = interview number.
